# Supplementary material for: Norway spruce postglacial recolonization of Fennoscandia
Source: Nat Commun. 2022 Mar 14;13:1333. doi: 10.1038/s41467-022-28976-4 (PMC8921311; doi:10.1038/s41467-022-28976-4)
Supplement: Supplementary file 1 — Supplementary information [file 41467_2022_28976_MOESM1_ESM.pdf]

Norway spruce postglacial recolonization of Fennoscandia

Nota *et al.*

Supplementary Note 1. Mitochondrial mh05 qPCR assay validation

Melting curve analysis was used because the 21-nucleotide deletion of the mh05 fragment is easily detectable based on its denaturing properties. The method implies amplifying DNA in a qPCR reaction. After the amplification is completed, the temperature is gradually increased from 60°C to 95°C. Depending on the DNA fragment size (more nucleotides will increase the melting temperature Tm) and nucleotide composition (A-T bonds require less energy to separate than G-C bonds) and because we are dealing with a 21-nucleotide deletion, the size difference will change the melting temperature. We tested the method using previously genotypes samples (3% agarose gels and/or Sanger sequenced). The melting curve for the shorter variant, haplotype A, showed a consistent melting peak between 73.2°C and 74°C, and the longer variant of haplotype B showed a melting peak between 75.4°C and 76°C. We found that modern DNA extracts needed to be diluted 100x to remove the effects of excessive salts and other compounds which increase the denaturing temperature of the PCR products during the melting curve analysis. Some melting temperatures (Tm) were still slightly outside the set bins, and these samples were Sanger sequenced (Supplementary Table 3). When both haplotypes were present in a single sample, the melting curve showed peaks easy to recognise with two melting peaks equally prevalent. When the concentration of one of the haplotypes was lower than the dominant variant, the melting peaks showed a tail in the melting range of the less common haplotype, which becomes prone to subjective interpenetration (Supplementary Figure 1). Of all the modern spruce samples, 32 were ambiguous, showing melting peaks outside the set bins and were therefore Sanger sequenced using the mh05_for and mh05_rev primers (Supplementary Table 3, sequences were only sequenced in the forward direction). All the sequenced products however, showed a clean product that could be used for genotyping. The electropherograms showed consistent minor peaks (results not shown) suggesting that there might be a gene duplication, likely in the nuclear genome. For this reason, all double peaks were genotyped according to the variant that was most dominant and in 33 out of 34 samples with a double peak, haplotype A was dominant. Based on the genotyping of 275 modern DNA extract, we set the detection thresholds for the ancient DNA to 73.2°C -74.4°C for haplotype A and 75.4°C to 76.4°C for haplotype B (Supplementary Figure 2).

We applied the qPCR melting cure assay to the ancient sediment sample instead of a high-throughput sequencing approach for the several reasons. The first reason is that amplification success is expected to be low for the two haplotypes and the complexity of the samples would be too low for high-throughput sequencing to provide good quality data. A second reason is that high-throughput sequencing is more sensitive than qPCR (theoretically a single copy of an amplicon can be detected) while qPCR requires a stronger signal to be visible after amplification. Finally, because complexity is very low in our data and it is coupled with a low success rate, we expected the number of false

positives to be much higher with high throughput sequencing than with qPCR. In this case it might be difficult to disentangle index-hopping, cross-contamination, with highly variable amplification success in samples. We chose not to use the standard p6-loop target which normally are used for plant metabarcoding because spruce and pine are difficult to differentiate from background contamination with these primers[1]. On the other hand, we know that haplotype A is a rare haplotype confined to western Fennoscandia and this reduces the risk of background contamination; if contamination occurs, it is more likely to be from haplotype B.

The *seda*DNA extractions were free from compounds that influenced Tm because inhibitor control template showed melting temperatures consistent with the expected value. For the ancient DNA analysis, depending on the lake, non-specific amplifications were observed in many samples. The melting temperatures of non-specific amplifications were typically higher than 78°C. For all qPCR reactions that contained ambiguous peaks, both haplotypes were considered as absent.

Supplementary Note 2. Nuclear SNP analyses

In total, we obtained 19,095,149 paired reads, with mean reads number per sample of 68,936±12,900. Due to the random nature of the method, and because of the large size of the spruce genome (20-Gb[2]) the SNP calling revealed a high proportion of missing data (e.g., SNP loci present in different proportions in different individuals). Due to the limited read coverage obtained per sample, the coverage per genotype after filtering was also relatively low overall, with a mean depth of 5.4±0.9X (±SD). We controlled that we had no informative missingness in our data by conducting all analyses (*Fst*, PCA, admixture; Supplementary Figures 3, 4, 5A-B, 6) using SNP loci with >50% or >25% missing data. All the results are consistent and robust to missing data. The *Fst*-values are presented in Supplementary Table 4 and Supplementary Table 5.

The PCAs including 10 individuals outside Fennoscandia are shown in Supplementary Figure 7. The PCA clearly shows a separation of the Carpathian and Alpine (Germany) populations along PC1 and PC2, respectively. The Russian-Baltic and *Picea obovata* separate along 3rd and 4th axes. These results are in line with results obtained in Chen *et al*.[3] and the same results were obtained using two missing data filters. We applied a lower filter for retaining genotypes than with the analysis for the samples from the Scandes. Instead of retaining only genotypes where the minor allele frequency was more than 0.03, we set this threshold to retaining all alternative alleles that occurred at least two times. We did this because we expect there to be more differences between the Fennoscandia samples

and the outgroups. With strict filtering, the alleles in the outgroup would very likely be filtered out, because they might be present in only two individuals.

Supplementary Note 3. The likelihood of DNA contamination in ancient samples

To control if the sediment samples were significantly enriched for haplotype A (Norway spruce) compared to a random detection, we simulated qPCR success as described below and the results showed that in all but three locations the discovery rate was higher than what is expected by change (Supplementary Table 6A). The simulations by 1000-year age slices for the six central Swedish lakes showed significant enrichment for all age intervals, except the oldest slice 10-11 cal. kyr BP and 4-5 cal. kyr BP (Supplementary Table 6B). The qPCR simulations were done by generating 5000 times a vector the length of the total numbers of PCR reactions performed (lab (207*8=1656, including controls: extraction negatives and PCR negatives) using binomial distribution with 0's (negative) and 1's (positive) and the *rbinom* function in R (R Core Team, 2020). The probability of a positive reaction was equal to possible false positive rates: PCR-negatives (1/192), extraction-negatives (1/272), overall detection (1176/54), and the site with no observations SR (1/48). We tested the false positive rate observed in Alsos *et al.*[8], using metabarcoding: 1.99% (mean) and 6.25% (upper). The p-values were assigned based on the "true sum of success" being more than 2*SD (one-sided) or more 3*SD (one-sided) of the simulated means. The value ($p<0.05$) was higher than 2 standard deviations and the value ($p<0.01$) was assigned if the value was more than 3 standard deviations. This showed that we would expect significantly more positive reactions in the large number of PCR and extraction controls analysed (Supplementary Table 6).

It is also unlikely that haplotype A distribution was due to contamination during subsampling, because samples from the lakes were collected in different expeditions, and core opening occurred at different locations, and in areas where haplotype A is absent or rare. It seems therefore very unlikely that contamination occurred at five different sites and exclusively at two specific age intervals (Supplementary Table 6B). No positive PCR reactions were obtained in the two Russian sites, and at one site in southern Finland, haplotype A was recovered in two different PCR reactions. If contamination occurred, more positive reactions would have been observed at these sites.

Supplementary Note 4. Additional discussion genetic continuity clonal trees and fossils

Our ancient DNA results show that spruce was present early and throughout the Holocene in Jämtland (central Sweden). The question of whether the old clonal trees could be survivors and contributed to

the next generations is only relevant if we consider them to be indeed ancient and originated before the massive spruce expansion in the late Holocene. If they are ancient, there is no evidence that they are different from the spruce forest. If they are more recently established (late Holocene) they must have replaced the population established in the early Holocene. In the latter scenario too, the possibility that trees survived the last glaciation cannot be excluded, but they did not contribute to the recolonization of Fennoscandia.

The necessity of proving the existence of a genetic link between the dated macrofossils and clonal spruce trees present today such as Old Tjikko, is a matter of debate. Our study, too, cannot prove genetic connectivity, and there is no way to do this with convincing genetic evidence yet (DNA amount in macrofossils is too little, contaminated, and damaged for being securely genotyped). Nevertheless, indirectly we strongly suggest there is such continuity, as haplotype A is present in Fennoscandia throughout the Holocene based on our sedaDNA results. This scenario rejects the hypothesis of a complete replacement of populations during the Holocene because haplotype A is not present outside Sweden and Norway, while in the Scandes is nearly fixed in some populations. One question may raise about the impact of our conclusions in case he sampled clonal trees above the treeline are instead young and not connected to the early Holocene colonisers. We argue that our conclusions would not change and do not need to be revised based on the following lines of reasoning imagining six possible scenarios:

A. Direct genetic link between dated macrofossils and trees – distinct western glacial refugia. In this case, the genetic variation present in the earliest established trees would not have changed and not be admixed with the variation of trees arriving later in the Holocene. Clonal trees would be grouping away from the surrounding forest. A signal would have been present, and we would have suggested that they originated from a separated glacial refugia but importantly did not contribute to the recolonization of Scandinavia.

B. Direct genetic link between dated macrofossils and trees – eastern glacial refugia. In this case, the genetic variation present in the earliest established trees would not have changed and not be admixed with genetic variation of trees arriving later in the Holocene. Clonal and forest trees would share a similar genetic history and would likely still be admixed between northern and southern clusters, because the area is a contact zone, and early colonisers could have arisen from admixed populations. In this case there would be no signal between clonal and forests trees, as detected in our study, and we therefore conclude a likely eastern origin and that there is no

separated glacial refugia, which importantly did not contribute to the recolonization of Scandinavia. However, it is not possible to exclude western refugia because genotypes in the refugia are the same.

C. No direct genetic link between dated macrofossils and trees but continuous presence – distinct western glacial refugia. In this case, the genetic variation present in the earliest established trees would be maintained and be at most admixed with genetic variation of trees arriving later in the Holocene. A signal would have been present and we would suggest that they originated from a separated glacial refugia, but importantly, we would still conclude they did not contribute to the recolonization of Scandinavia.

D. No direct genetic link between dated macrofossils and trees but continues presence – eastern glacial refugia. In this case, the genetic variation present in the earliest established trees would be maintained and be at most admixed with genetic variation of trees arriving later in the Holocene. Clonal trees and forest trees would share a similar genetic history and would likely be admixed because the area is in a contact zone. In this case there would be no difference between clonal and forests trees, as detected in our study, and we would conclude eastern origin and that there is no separated glacial refugia, which importantly did not contribute to the recolonization of Scandinavia. But we could not exclude the possibility that few trees survived in the west because genotypes in the refugia are the same.

E. No direct genetic link between dated macrofossils and trees without continues presence – distinct western glacial refugia. In this scenario there were periods where the clonal populations were not present and were replaced one or multiple times during the Holocene. In this case, the spruce populations would be young, and resemble the observed data, with no separation of the clonal and surrounding forest. It would not be possible to detect the distinct western refugia because the connectivity is gone.

F. No direct genetic link between dated macrofossils and trees without continues presence – eastern glacial refugia. In this scenario there were periods where the clonal populations were not present and were replaced one or multiple times during the Holocene. In this case, the spruce populations would be young, and resemble the observed data, with no separation of the clonal and surrounding forest. Not possible to exclude western refugia because genotypes in the refugia are the same.

Scenarios A and C can be rejected because there is no signal of a distinct western genotype. Scenarios B and D would both produce the results observed in our study, and both would lead to the same conclusions: early colonisation from east or east and south with no rejection of a western refugia because trees might belong to the same two widespread genetic clusters. Scenarios E and F would produce the genetic structure observed in our study with nuclear markers, but it would not agree with the distribution of the mitochondrial haplotype A we found, because there would be no source area for this haplotype. Therefore, according to our data B and D are the most likely scenarios and since we believe there is genetic continuities between dated macrofossils and trees, scenario B is in our view more likely than D. Nevertheless, since both B and D are possible, it is even likely a combination of the two. The most important thing is that our conclusions hold regardless genetic connectivity between fossils and trees, and regardless genetic continuity due to sexual reproduction occurred during the Holocene.

Supplementary Note 5. Inhibition control on *sed*aDNA extracts

All *sed*aDNA samples were assessed for inhibition, an overview of the dilution factor needed for the qPCR not to be inhibited based on the spike in template (Supplementary Figure 8). See the main text and Capo *et al.*[4] for more information on the methodology.

Supplementary Note 6. Sanger sequencing preparations of sedaDNA samples

We redesigned primers for amplifying the *mh05* fragment in ancient DNA samples compared to the primers used by Parducci *et al.*[5] and we used primer set *mh05short* for amplification of shorter mh05 fragments in ancient lakes which resulted in products in the range between 64 and 85 base pairs including the primer binding sites (Supplementary Table 7). Such small fragments cannot be accurately Sanger sequenced, therefore we ligated Illumina adapters to 1 µL of qPCR product according to Carøe *et al.*[6] except that the MinElute clean-up was performed before the fill-in step. The adapter ligated qPCR product was amplified for 10 cycles in PCR using the primer pair *mh05shortFOR* and qPCR_IS8[7]. The PCR contained 1X QIAGEN Multiplex PCR master mix, 0.2 µM of each primer in 20 µL reaction volume. Supplementary Table 8 shows the Sanger sequencing results. In five cases Sanger sequences were of bad quality and could not be used for genotyping. Non-specific sequences could not be identified based on Blast.

Supplementary Table 1. Overview of the samples used for ancient DNA analysis.

Location	Name	N samples	Age range cal. kry BP	Elevation	Sediment type	Reference	Coring year
West Finnish Lapland	NWF	3	42		Fluvial peat	8,9	2012
East-Finnish Lapland	EFL-Salla	1	8.6#		Fluvial peat	Not published	2016
Finnish Lapland	Kou	1	42.5$		Fluvial peat	Not published	2016
North-east European Russia	NER	10	8.5-6		Peat	5	2012
Southern European Russia	SR	6	13.5-4.5		Lake	Not published	2010
Southern Finland	SF	12	6.2-0.2		Lake	10	2011
Central Sweden	CS-KL	9	10.4-0.9	540	Lake	8	2008
Central Sweden	CS-ZF10	18	8.9-1.2	573	Lake	4	2018
Central Sweden	CS-ZF11	14	9.3-0.2	564	Lake	4	2018
Central Sweden	CS-ZF18	16	9.8-0.4	735	Lake	4	2018
Central Sweden	CS-ZF19	11	9.3-0.3	812	Lake	4	2018
Central Norway	CN-RD	9	10.4-0.9	330	Lake	8	2008
Southwest Sweden	SWS	12	15.3-11		Lake/peat	11	2011
Southeast Sweden*	SES	2x8	13.8-10.5		Lake/peat	12, 13	2015

* Two cores, # raw radiocarbon date (9450+/-60BP), $ raw radiocarbon date 43200+/-1600BP

Supplementary Table 2. sampling location modern spruce populations.

Location	Country	Regio	Coordinates	Population	Ind. (n)	Altitude (mean)	Dated tree – cal. yr BP
Fulufjället	Sweden	Dalarna	61.63N, 12.67E	Clonal	20	897-926 (915)	Old Tjikko – 9550
			61.64N, 12.71E	Forest	27	<862	
Städjan	Sweden	Dalarna	61.90N, 12.87E	Clonal	21	899-948 (910)	Old Linnegranen – 4420
			61.89N, 12.71E	Forest	26	<898	
Härjehågna	Sweden	Dalarna	61.72N, 12.15E	Clonal	20	921-986 (958)	Gunnar Samuelsson spruce (GS spruce)– 6310
							Unnamed tree – 7890
							Unnamed tree – 4800
Sälen	Sweden	Dalarna	61.17N, 13.13E	Clonal	20	778-826 (808)	Lundqvists gran – 7940
Hårdeggen	Sweden	Jämtland	63.20N, 12.43E	Mixed	20	634-795 (688)	
Getryggen	Sweden	Jämtland	63.17N, 12.35E	Clonal	5	765-830 (793)	Old Pompe – 6400
Snåsahögarna*	Sweden	Jämtland	63.22N, 12.42E	Forest	20	549-685 (621)	
Åreskutan	Sweden	Jämtland	63.40N, 13.05E	Forest	20	613-763 (678)	Old Molly* – 6400
Lill-Skarven	Sweden	Härjedalen	62.55N, 12.35E	Clonal	20	880-975 (994)	
Sonfjället	Sweden	Härjedalen	62.29N, 13.57E	Forest	20	-	Old Rasmus* – 9480
							Old Risa* – 2340
							Old Victor* – 2340
Drevfjället	Norway		61.77N, 12.08E	Clonal	27	888-1004 (939)	

* Not sampled

Supplementary Table 3. Overview of inconclusive qPCR multicurve genotypes from modern Norway spruce samples and double melting curves.

			mh05 Genotype		
**Sample**	**Tree type**	**Melting curve**	**major melting curve**	**Sanger sequencing**	**Final genotype**
FU_Old_Tjikko_1	Clonal	A	A	A	A
FU_5	Clonal	?	?	A	A
FU_7	Clonal	?	?	B	B
FU_25	Forest	?	A	A	A
FU_33	Forest	A	A	A	A
FU_40	Forest	A/B	A	-	A
ST_Old_Linnegranen	Clonal	A/B	A	A	A
ST_23	Clonal	A/B	A	-	A
ST_25	Clonal	A/B	A	-	A
ST_26	Clonal	A/B	A	-	A
ST_1	Forest	A	A	A	A
ST_3	Forest	?	?	A	A
ST_4	Forest	B	B	B	B
ST_5	Forest	?	?	A	A
ST_6	Forest	A	A	A	A
ST_7	Forest	B	B	B	B
ST_8	Forest	A	A	A	A
ST_10	Forest	A/B	A	-	A
ST_11	Forest	A/B	A	-	A
DR_3	Clonal	A/B	A	-	A
DR_6	Clonal	A	A	A	A
DR_7	Clonal	?	?	B	B
DR_8	Clonal	?	?	B	B
DR_22	Clonal	?	?	B	B
HH_3	Clonal	?	?	B	B
HH_5	Clonal	A	A	A	A
HH_6	Clonal	B	B	B	B
HH_9	Clonal	A	A	A	A
Sal_1	Clonal	B	B	B	B
Sal_7	Clonal	?	?	A	A
Sal_9	Clonal	A/B	A	-	A
Sal_10	Clonal	A/B	A	-	A
Sal_19	Clonal	A/B	A	-	A
Hår_7	Forest	A/B	B	-	B
Hår_8	Forest	B	B	B	B
Hår_18	Forest	A/B	A	-	A
Get_Old_Pompe	Clonal	B	B	B	B
SN_5	Forest	A/B	A	A	A
SN_7	Forest	A	A	A	A
SN_8	Forest	A	A	A	A
SN_11	Forest	A/B	A	-	A
SN_14	Forest	A/B	A	-	A
År_1	Forest	A/B	A	-	A
År_5	Forest	A/B	A	-	A
År_7	Forest	A/B	A	A	A
År_11	Forest	A/B	A	-	A
År_14	Forest	A/B	A	A	A
År_19	Forest	A	A	A	A
År_20	Forest	A/B	A	-	A
Lsk_5	Clonal	?	?	A	A
Lsk_6	Clonal	A/B	A	-	A
Lsk_8	Clonal	A/B	A	A	A
Lsk_9	Clonal	A/B	A	-	A
Lsk_10	Clonal	A/B	A	-	A
Lsk_13	Clonal	A/B	A	-	A
Lsk_15	Clonal	A/B	A	-	A
Lsk_19	Clonal	A/B	A	-	A
Lsk_20	Clonal	A/B	A	-	A
Son_1	Forest	A/B	A	-	A
Son_2	Forest	A	A	A	A
Son_3	Forest	A/B	A	-	A
Son_4	Forest	A/B	A	-	A
Son_5	Forest	A/B	A	-	A
Son_6	Forest	A/B	A	-	A
Son_7	Forest	A/B	A	A	A
Son_8	Forest	A/B	A	A	A
Son_10	Forest	A/B	A	-	A
Son_12	Forest	A/B	A	-	A
Son_15	Forest	A/B	A	-	A
Son_19	Forest	A/B	A	-	A

Supplementary Table 4. Fst-values loci with no missing data filter.

Population	Åre	Snå	Hår	Get	Lsk	Son	Stä-C	Stä-F	Här	Dre	Ful-C	Ful-F	Säl
Åreskutan		0.682	1.000	0.408	0.146	0.000	0.000	0.000	0.000	0.000	0.000	0.000	0.000
Snåsahögarna	-0.001		0.703	0.115	0.003	0.000	0.000	0.001	0.000	0.000	0.000	0.000	0.000
Hårdeggen	-0.009	-0.001		0.848	0.019	0.190	0.000	0.001	0.000	0.000	0.000	0.000	0.000
Getryggen	0.001	0.006	-0.004		0.222	0.633	0.286	0.088	0.019	0.000	0.005	0.122	0.024
Lill-Skarven	0.003	0.008	0.005	0.003		0.042	0.008	0.000	0.000	0.000	0.203	0.397	0.000
Sonfjället	**0.010**	**0.012**	0.002	-0.001	0.004		0.000	0.000	0.005	0.005	0.270	0.024	0.007
Städjan-C	**0.015**	**0.014**	**0.018**	0.002	0.006	0.010		0.087	0.057	0.039	0.031	0.004	0.001
Städjan-F	**0.019**	**0.009**	**0.011**	0.007	**0.012**	**0.011**	0.004		0.023	0.065	0.566	0.000	0.000
Härjehågna	**0.022**	**0.018**	**0.022**	0.009	**0.008**	**0.007**	0.004	0.005		0.792	0.983	0.234	0.996
Drevfjället	**0.020**	**0.021**	**0.025**	0.014	**0.010**	0.008	0.005	0.005	-0.002		0.242	0.300	0.498
Fulufjället-C	**0.016**	**0.012**	**0.010**	0.010	0.002	0.002	0.004	-0.001	-0.004	0.002		0.038	0.842
Fulufjället-F	**0.014**	**0.015**	**0.011**	0.005	0.001	0.005	0.006	**0.008**	0.002	0.002	0.004		0.019
Sälen	**0.025**	**0.025**	**0.026**	0.009	**0.013**	0.007	**0.007**	**0.010**	-0.006	0.000	-0.002	0.005	

Fst-values calculated using the StAMPP package, using[14], see materials and methods main paper

Supplementary Table 5. Fst-values loci with maximum 25% missing data.

Population	Åre	Snå	Hår	Get	Lsk	Son	Stä-C	Stä-F	Här	Dre	Ful-C	Ful-F	Säl
Åreskutan		0.075	0.101	0.954	0.000	0.000	0.000	0.000	0.000	0.000	0.000	0.000	0.000
Snåsahögarna	0.001		0.178	0.861	0.004	0.000	0.000	0.000	0.000	0.000	0.000	0.000	0.000
Hårdeggen	0.001	0.001		0.585	0.000	0.000	0.000	0.000	0.000	0.000	0.000	0.000	0.000
Getryggen	-0.004	-0.003	0.000		0.663	0.258	0.793	0.095	0.078	0.015	0.034	0.048	0.019
Lill-Skarven	**0.003**	0.003	**0.004**	-0.001		0.347	0.000	0.000	0.000	0.000	0.000	0.001	0.000
Sonfjället	**0.005**	**0.004**	**0.007**	0.002	0.000		0.000	0.000	0.000	0.000	0.000	0.001	0.000
Städjan-C	**0.009**	**0.007**	**0.009**	-0.002	**0.004**	**0.004**		0.003	0.071	0.000	0.000	0.000	0.003
Städjan-F	**0.009**	**0.009**	**0.009**	0.003	**0.004**	**0.004**	0.002		0.000	0.000	0.321	0.006	0.000
Härjehågna	**0.014**	**0.011**	**0.018**	0.004	**0.006**	**0.005**	0.001	**0.003**		0.449	0.079	0.000	0.566
Drevfjället	**0.015**	**0.012**	**0.016**	0.006	**0.007**	**0.005**	**0.004**	**0.002**	0.000		0.833	0.667	0.539
Fulufjället-C	**0.010**	**0.007**	**0.011**	0.005	**0.003**	**0.003**	**0.002**	0.000	0.001	-0.001		0.981	0.092
Fulufjället-F	**0.011**	**0.010**	**0.011**	0.005	**0.003**	**0.003**	**0.003**	0.002	**0.003**	0.000	-0.002		0.066
Sälen	**0.016**	**0.015**	**0.019**	0.005	**0.007**	**0.006**	0.002	**0.004**	0.000	0.000	0.001	0.001	

Fst-values calculated using the StAMPP package, using[14] see materials and methods main paper.

Supplementary Table 6. Values for differences between true sum of positive results and of 3*SD of 5000 simulated sums using 6 false positive rates.

			Mean difference from the true sum											
False positive rate			*0.0625*[1]		*0.0478*		*0.0199*[1]		*0.0125*		*0.#052*		*0.0037*	
			Simulated means + and – 3SD											
Sites	N-sample	N pos	-	+	-	+	-	+	-	+	-	+	-	+
CN-RD	9	2	-3.66	8.62	-4.06	6.64	-4.17	3.01	-3.96	1.75	-3.41	**0.10**[*]	-3.28	**-0.19**[**]
CS-ZF10	18	5	-4.75	12.76	-5.92	9.08	-7.17	2.93	-7.23	0.80	-6.87	**-1.71**[**]	-6.62	**-2.34**[**]
CS-ZF11	14	5	-5.59	9.67	-6.50	6.79	-7.28	1.74	-7.17	**-0.03**[**]	-6.60	**-2.34**[**]	-6.52	**-2.65**[**]
CS-KL	9	6	-7.62	4.65	-7.97	2.66	-8.13	**-1.04**[**]	-7.93	**-2.23**[**]	-7.41	**-3.90**[**]	-7.29	**-4.16**[**]
CS-ZF18	16	9	-9.17	7.19	-10.34	4.17	-11.17	**-1.67**[**]	-11.14	**-3.66**[**]	-10.72	**-6.03**[**]	-10.59	**-6.46**[**]
CS-ZF19	11	6	-7.33	6.34	-7.88	4.01	-8.21	**-0.26**[**]	-8.02	**-1.84**[**]	-7.51	**-3.68**[**]	-7.40	**-3.94**[**]
SWS-ATT	12	9	-7.75	10.83	-9.43	6.89	-11.22	**-0.06**[**]	-11.18	**-2.61**[**]	-10.89	**-5.47**[**]	-10.74	**-6.03**[**]
SES-HA1	8	1	-2.83	8.77	-3.11	7.02	-3.06	3.53	-2.84	2.44	-2.36	0.99	-2.23	0.71
SES-HA2	8	2	-3.85	7.90	-4.02	5.96	-4.12	2.65	-3.87	1.50	-3.35	**-0.03**[**]	-3.23	**-0.31**[**]
SF	12	2	-3.12	11.08	-3.76	8.62	-4.28	4.01	-4.05	2.48	-3.55	0.47	-3.43	**0.15**[*]
EFL-Sal	1	1	-3.02	7.04	-3.14	5.49	-3.00	2.91	-2.70	1.90	-2.22	0.72	-2.05	**0.39**[*]
EFL_Kou	1	1	-2.58	1.60	-2.39	1.11	-2.02	0.34	-1.84	**0.05**[*]	-1.53	**-0.40**[**]	-1.51	**-0.43**[**]
NWF	3	5	-7.05	**0.08**[*]	-6.98	**-0.84**[**]	-6.62	**-2.44**[**]	-6.37	**-3.01**[**]	-5.90	**-3.88**[**]	-5.77	**-4.07**[**]
SR	6	0	-1.60	2.65	-1.46	2.21	-1.02	1.34	-0.83	1.02	-0.54	0.62	-0.51	0.58
NER	10	0	-1.53	11.58	-1.99	9.38	-2.19	5.40	-1.97	3.98	-1.45	2.23	-1.33	1.93
Ext-NEG	24	1	**1.60**[**a]	22.35	**-0.49**[*a]	17.70	-2.85	9.19	-3.24	6.42	-2.94	2.94	-2.84	2.38
PCR-NEG	34	1	**4.04**[**a]	27.85	**1.04**[**a]	21.88	-2.53	11.37	-3.15	7.84	-3.12	3.72	-2.93	2.92

			Mean difference from the true sum											
False positive rate			*0.0625*[1]		*0.0376*		*0.0199*[1]		*0.0208*		*0.0052*		*0.0037*	
			Simulated means + and – 3SD											
kyr BP	N-sample	N-pos.	-	+	-	+	-	+	-	+	-	+	-	+
0-1	6	3	-5.01	4.99	-5.19	3.57	-4.95	0.85	-4.73	**-0.05**[**]	-4.21	**-1.33**[**]	-4.05	**-1.63**[**]
1-2	7	2	-3.85	6.91	-4.16	5.26	-4.03	2.24	-3.80	1.13	-3.26	**-0.22**[**]	-3.15	**-0.46**[**]
2-3	11	8	-9.42	4.41	-9.84	1.95	-10.11	**-2.44**[**]	-10.02	**-3.80**[**]	-9.56	**-5.58**[**]	-9.37	**-6.01**[**]
3-4	7	2	-3.95	6.92	-4.07	5.15	-4.02	2.25	-3.79	1.18	-3.30	**-0.14**[**]	-3.14	**-0.46**[**]
4-5	7	1	-2.88	7.94	-3.12	6.32	-3.06	3.29	-2.77	2.18	-2.29	0.82	-2.19	0.60
5-6	7	2	-3.95	6.98	-4.12	5.31	-3.96	2.17	-3.80	1.18	-3.29	**-0.16**[**]	-3.14	**-0.45**[**]
6-7	9	5	-6.66	5.66	-7.05	3.70	-7.13	0.01	-6.90	**-1.29**[**]	-6.44	**-2.87**[**]	-6.29	**-3.17**[**]
7-8	10	6	-7.42	5.35	-7.95	3.23	-8.18	**-0.67**[**]	-7.96	**-2.05**[**]	-7.44	**-3.81**[**]	-7.36	**-4.05**[**]
8-9	6	2	-4.06	6.13	-4.18	4.60	-3.96	1.86	-3.75	0.94	-3.18	**-0.37**[**]	-3.09	**-0.53**[**]
9-10	5	2	-4.04	5.05	-4.07	3.72	-3.86	1.44	-3.61	**0.61**[*]	-3.15	**-0.46**[**]	-2.98	**-0.74**[**]
10-11	2	1	-1.95	3.96	-1.78	3.27	-1.36	1.99	-1.16	1.58	-0.75	0.90	-0.68	0.80

* Sum significantly lower than observed (p<0.05), ** Sum significantly lower than observed (p<0.01), [*a] Sum significantly higher than observed (p<0.05), [**a] Sum significantly higher than observed (p<0.01), [8] Alsos *et al.* See "The likelihood of DNA contamination in ancient samples" for acquiring p-values.

Supplementary Table 7. Overview of the mh05 primer pairs used.

Primer name	Sequence 5'->3'	Product Size	Annealing Tm	Reference
mh05_for	GGGAGTCAGCGAAAGAAGTAAG	241-262	57°C	15
mh05_rev	AGTCTCAGAGCCAGAAGCAG			
mh05int_for	CCCCTAAGTAAGTAAACCTCTA	120-141		1
mh05int_rev	TCAGAGCCAGAAGCAGATTCAC			
mh05shortFOR	ACAAGAGCTGTAGACCTTAGGAA	64-85	55°C*, 60°C^{+}	This study
mh05shortREV	ATTCACCCGCAGCAAAGG			

For ancient DNA* and for modern DNA+ genotyping.

Supplementary Table 8. Overview of Sanger sequencing results.

Sample	Sequence direction	qPCR genotype	Sanger sequence	Quality
NER3_R#1	Reverse	B	?	Low
NER09_R#1	Reverse	Non-Specific	Non-Specific	high
NER10_R#7	Reverse	?	?	Low
SF10_R#2	Reverse	B	B	high
SF10_F#2	Forward	B	B	high
SF12_R#3	Reverse	?	?	Low
RD04_R#1	Reverse	B	?	Low
RD05_R#3	Reverse	Non-Specific	?	Low
RD07_R#4	Reverse	A	A	Relatively high
KL03_F#5	Forward	A	A	high
KL03_R#5	Reverse	A	A	high
KL04_F#1	Forward	B	B	high
KL04_R#1	Reverse	B	B	Relatively Good
KL06_R#6	Reverse	?	?	Low
KL07_R#1	Reverse	A	A	Relatively Good
ZF10-14_R#2	Reverse	A	?	Mixed sequence
ZF10-14_F#2	Forward	A	?	Mixed sequence
NWF02_R#3	Reverse	A	?	Low

qPCR repeat that was sequenced.

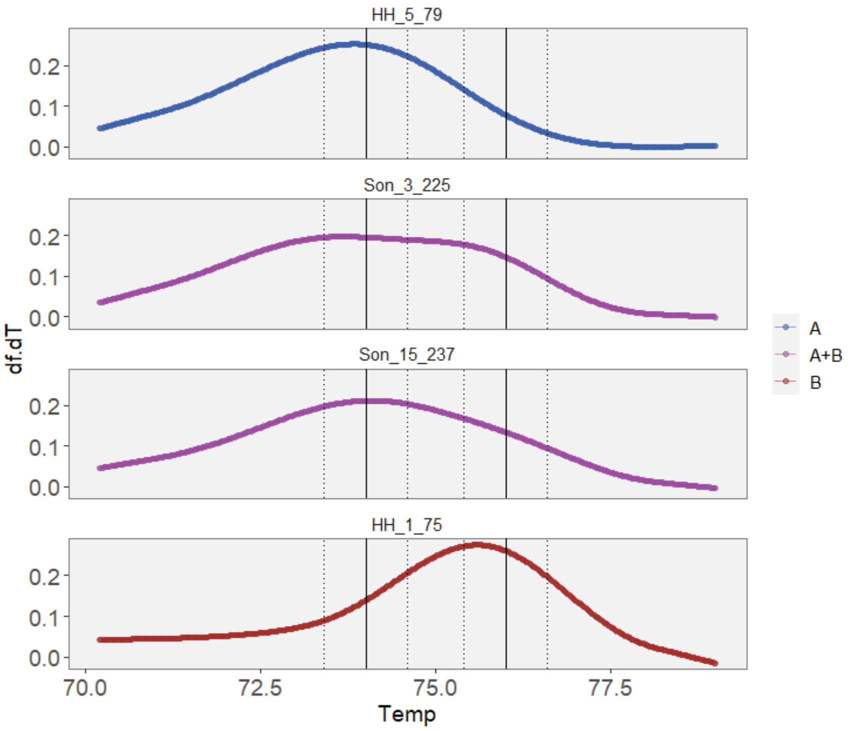

Supplementary Figure 1. Example of double peaks indicating two copies of the same locus. In sample Son_3 both peaks are clearly present, while in Son_15 the two peaks are less marked, however, the tail into hap B range is visible. In both cases, haplotype A was scored as the true and dominant mitochondrial variant.

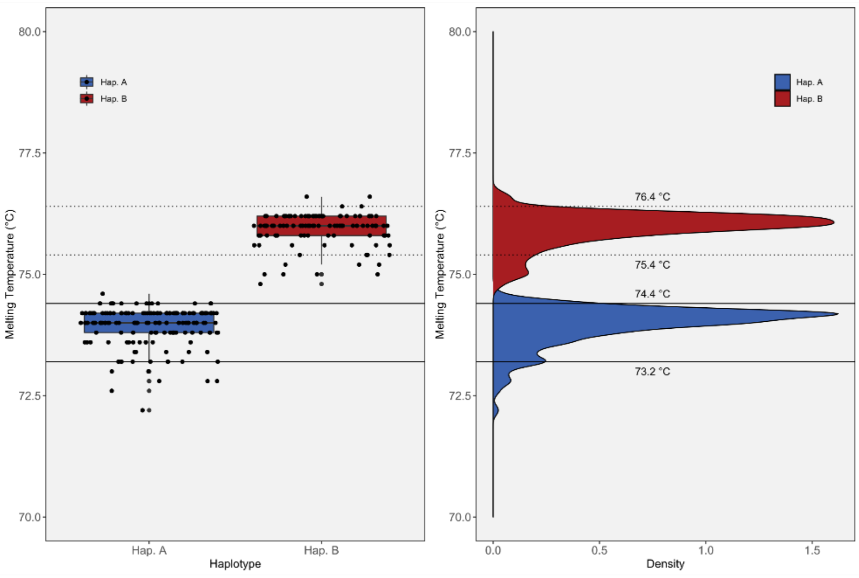

Supplementary Figure 2. Overview of 275 modern DNA melting curve genotyping samples (haplotypes A in blue, and haplotype B in red). The graphs show 14 samples with unconclusive melting peaks that were excluded. Data presented as median (50% quartile), and minima and maxima (25% quartiles).

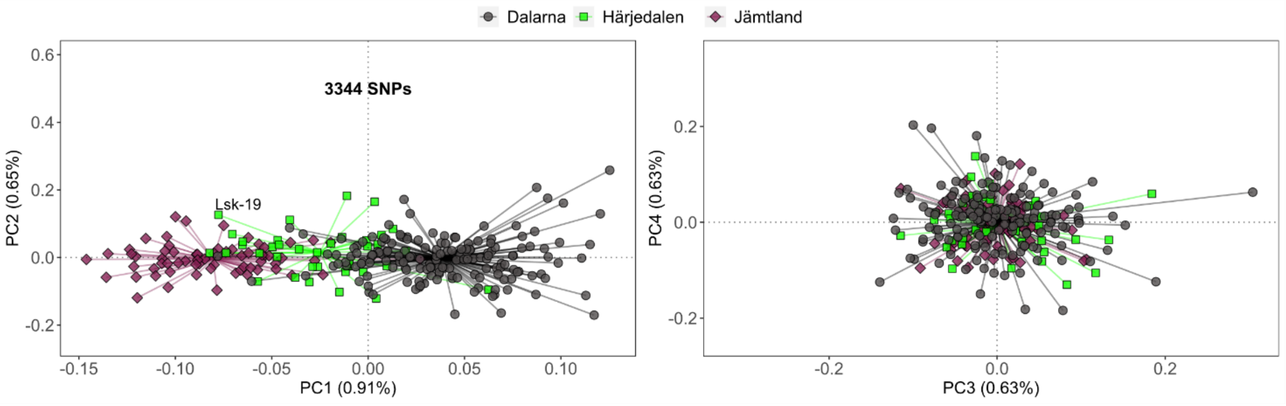

Supplementary Figure 3. PCA with more stringent filtering shows the same structure as observed when all the data is included (only results of >25% missing data is shown, and there were no differences between 50% and 25% missing data).

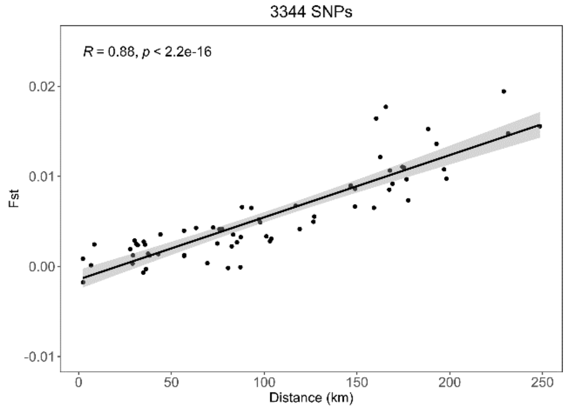

Supplementary Figure 4. Isolation by distance using the most stringent filtered dataset. Filtered and non-filtered data showed the same pattern. Fitting Generalized Linear Model (GLM) was used to produce confidence interval (geom_smooth(method="glm")). Pearson correlation (two-sided, stat_cor()).

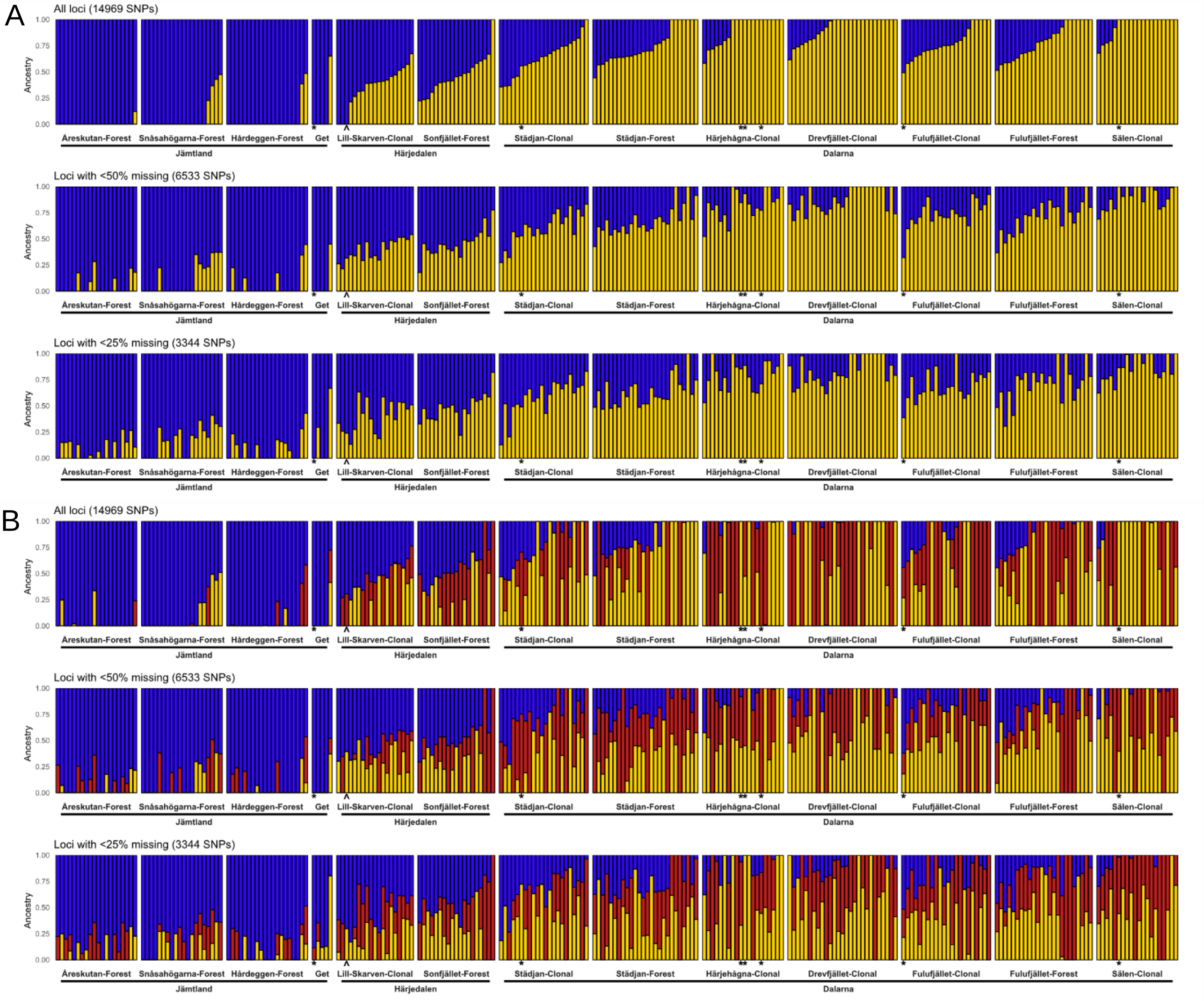

Supplementary Figure 5. Admixture K=2 (A), and k=3 (B), spruce populations are ordered according to latitude. The samples are sorted according to the ancestry component when all loci are included. The bars of dated clonal trees are indicated with an asterisk, and the outlier sample observed in the PCA is indicated with the symbol ^. The overall pattern of the admixture is not influenced by the number of SNPs used. Only a slight differentiation is observed in the proportion of ancestry in individuals between the different filtered datasets.

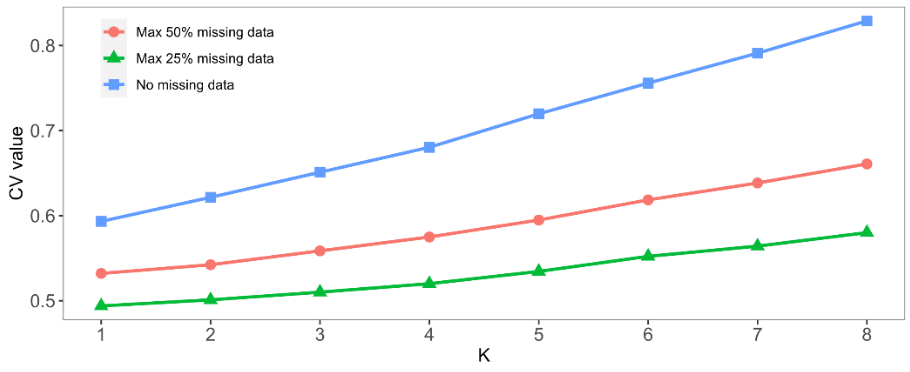

Supplementary Figure 6. Cross-validation plot showing k=0 to k=8. The value of K with the lowest variation is K=1, K=2 and 3 are only slightly higher. Similar results are obtained with all datasets.

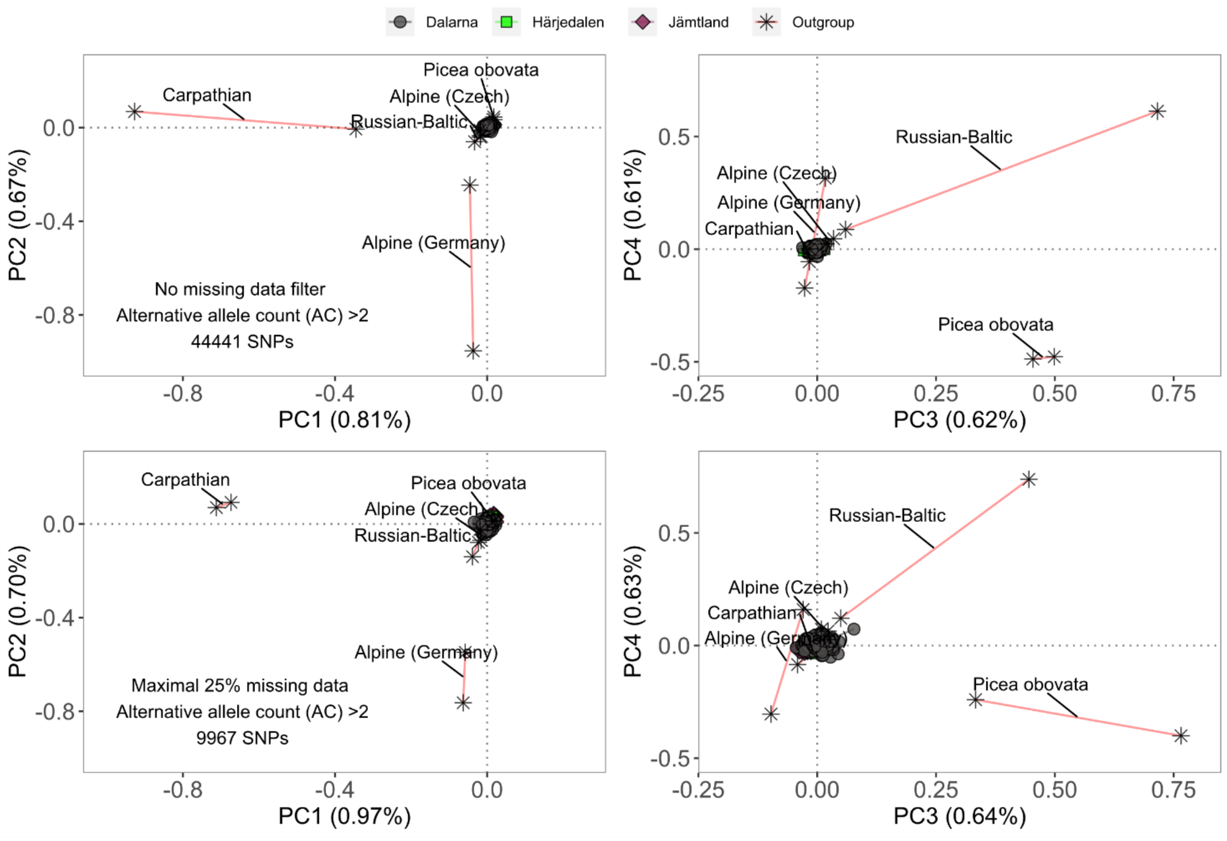

Supplementary Figure 7. PC1-2 and PC 3-4 displayed for two different missing data filters, the number of SNPs used is given in the plots. Carpathian and Alpine (Germany) individuals separate in the first and second PC axes, respectively. The Russian-Baltic and Picea obovata are separating from the Fennoscandia samples in the thirds and fourth axes.

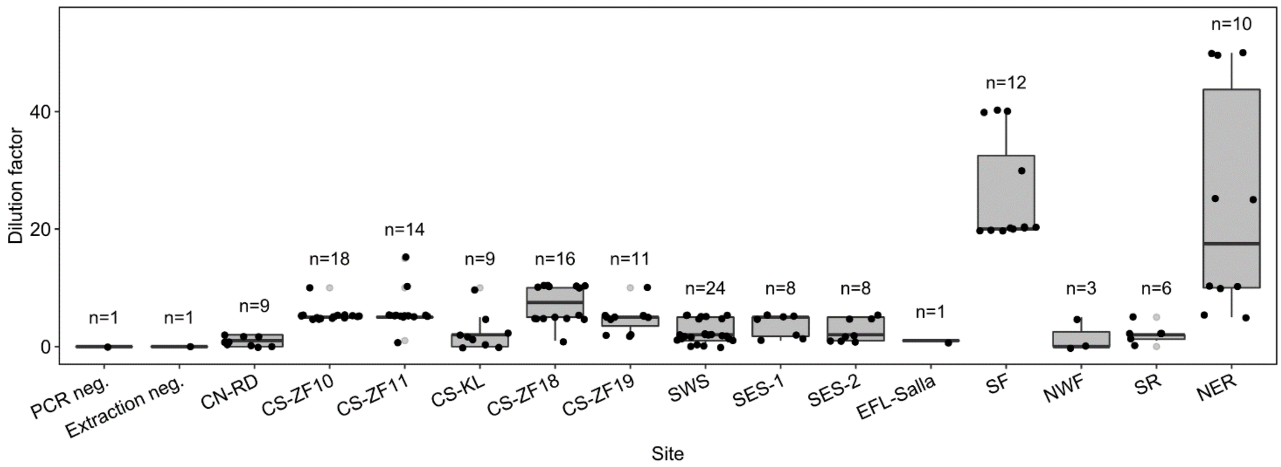

Supplementary Figure 8. Boxplots of dilutions required to prevent inhibition. Generally, a dilution 0-10X was sufficient, except for samples from southern Finland (SF) and northern-east Russia (NER). Data presented as median (50% quartile), and minima and maxima (25% quartiles).

Supplementary references

1. Alsos, I. G., Sjögren, P., Brown, A. G., Gielly, L., Merkel, M. K. F., Paus, A., Lammers, Y., Edwards, M. E., Alm, T., Leng, M., Goslar, T., Langdon, C. T., Bakke, J., & van der Bilt, W. G. M. Last Glacial Maximum environmental conditions at Andøya, northern Norway; evidence for a northern ice-edge ecological "hotspot". *Quaternary Science Reviews* **239**, 106364 (2020).
2. Nystedt, B., Street, N. R., Wetterbom, A., Zuccolo, A., Lin, Y. C., Scofield, D. G., Vezzi, F., Delhomme, N., Giacomello, S., Alexeyenko, A., Vicedomini, R., Sahlin, K., Sherwood, E., Elfstrand, M., Gramzow, L., Holmberg, K., Hällman, J., Keech, O., Klasson, L., … Jansson, S. The Norway spruce genome sequence and conifer genome evolution. *Nature* **497**, 579–584 (2013)
3. Chen, J., Li, L., Milesi, P., Jansson, G., Berlin, M., Karlsson, B., Aleksic, J., Vendramin, G. G., & Lascoux, M. Genomic data provide new insights on the demographic history and the extent of recent material transfers in Norway spruce. *Evolutionary Applications* **12**, (2019)
4. Capo, E., Ninnes, S., Domaizon, I., Bertilsson, S., Bigler, C., Wang, X.-R., Bindler, R., & Rydberg, J. Landscape Setting Drives the Microbial Eukaryotic Community Structure in Four Swedish Mountain Lakes over the Holocene. *Microorganisms* 9, **355** (2021).
5. Parducci, L., Edwards, M. E., Bennett, K. D., Alm, T., Elverland, E., Tollefsrud, M. M., Jørgensen, T., Houmark-Nielsen, M., Larsen, N. K., Kjær, K. H., Fontana, S. L., Alsos, I. G., & Willerslev, E. Response to comment on "Glacial survival of boreal trees in Northern Scandinavia." *Science* **338**, 9–10 (2012).
6. Carøe, C., Gopalakrishnan, S., Vinner, L., Mak, S. S. T., Sinding, M. H. S., Samaniego, J. A., Wales, N., Sicheritz-Pontén, T., & Gilbert, M. T. P. Single-tube library preparation for degraded DNA. *Methods in Ecology and Evolution* **9**, 410–419 (2018).
7. Meyer, M., & Kircher, M. Illumina sequencing library preparation for highly multiplexed target capture and sequencing. *Cold Spring Harbor Protocols* **5**, pdb.prot5448 (2010).
8. Parducci, L., Väliranta, M., Sakari Salonen, J., Ronkainen, T., Matetovici, I., Fontana, S. L., Eskola, T., Sarala, P., & Suyama, Y. Proxy comparison in ancient peat sediments: Pollen, macrofossil and plant DNA. *Philosophical Transactions of the Royal Society B: Biological Sciences* **370**, 20130382 (2015).
9. Sarala, P., Väliranta, M., Eskola, T., & Vaikutiené, G. First physical evidence for forested environment in the Arctic during MIS 3. *Scientific Reports* **6**, 29054 (2016).
10. Ojala, A. E. K., & Alenius, T. 10000 years of interannual sedimentation recorded in the Lake Nautajärvi (Finland) clastic–organic varves. *Palaeogeography, Palaeoclimatology, Palaeoecology* **219**, 285–302 (2005).

11. Wohlfarth, B., Luoto, T. P., Muschitiello, F., Väliranta, M., Björck, S., Davies, S. M., Kylander, M., Ljung, K., Reimer, P. J., & Smittenberg, R. H. Climate and environment in southwest Sweden 15.5–11.3 cal. ka BP. *Boreas* **47**, 687–710 (2018).

12. Wohlfarth, B., Muschitiello, F., L. Greenwood, S., Andersson, A., Kylander, M., Smittenberg, R. H., Steinthorsdottir, M., Watson, J., & Whitehouse, N. J. Hässeldala – a key site for Last Termination climate events in northern Europe. *Boreas* **46**, 143–161 (2017).

13. Parducci, L., Alsos, I. G., Unneberg, P., Pedersen, M. W., Han, L., Lammers, Y., Salonen, J. S., Väliranta, M. M., Slotte, T., & Wohlfarth, B. Shotgun environmental DNA, pollen, and macrofossil analysis of lateglacial lake sediments from southern Sweden. *Frontiers in Ecology and Evolution* **7**, 189 (2019).

14. Weir, B. S., & Cockerham, C. C. Estimating F-Statistics for the Analysis of Population Structure. *Evolution* **38**, 1358 (1984).

15. Jeandroz, S., Bastien, D., Chandelier, A., Du Jardin, P., & Favre, J. M. A set of primers for amplification of mitochondrial DNA in Picea abies and other conifer species. *Molecular Ecology Notes* **2**, 389–392 (2002).
