# Supplementary material for: Norway spruce postglacial recolonization of Fennoscandia
Source: Nat Commun. 2022 Mar 14;13:1333. doi: 10.1038/s41467-022-28976-4 (PMC8921311; doi:10.1038/s41467-022-28976-4)
Supplement: Supplementary file 3 — Reporting Summary [file 41467_2022_28976_MOESM3_ESM.pdf]

nature portfolio

Corresponding author(s): Laura Parducci

Last updated by author(s): Feb 5, 2022

Reporting Summary

Nature Portfolio wishes to improve the reproducibility of the work that we publish. This form provides structure for consistency and transparency in reporting. For further information on Nature Portfolio policies, see our Editorial Policies and the Editorial Policy Checklist.

Statistics

For all statistical analyses, confirm that the following items are present in the figure legend, table legend, main text, or Methods section.

n/a	Confirmed	
☐	☒	The exact sample size (*n*) for each experimental group/condition, given as a discrete number and unit of measurement
☐	☒	A statement on whether measurements were taken from distinct samples or whether the same sample was measured repeatedly
☒	☐	The statistical test(s) used AND whether they are one- or two-sided<br>*Only common tests should be described solely by name; describe more complex techniques in the Methods section.*
☒	☐	A description of all covariates tested
☒	☐	A description of any assumptions or corrections, such as tests of normality and adjustment for multiple comparisons
☐	☒	A full description of the statistical parameters including central tendency (e.g. means) or other basic estimates (e.g. regression coefficient) AND variation (e.g. standard deviation) or associated estimates of uncertainty (e.g. confidence intervals)
☐	☒	For null hypothesis testing, the test statistic (e.g. *F*, *t*, *r*) with confidence intervals, effect sizes, degrees of freedom and *P* value noted<br>*Give P values as exact values whenever suitable.*
☒	☐	For Bayesian analysis, information on the choice of priors and Markov chain Monte Carlo settings
☒	☐	For hierarchical and complex designs, identification of the appropriate level for tests and full reporting of outcomes
☒	☐	Estimates of effect sizes (e.g. Cohen's *d*, Pearson's *r*), indicating how they were calculated

Our web collection on statistics for biologists contains articles on many of the points above.

Software and code

Policy information about availability of computer code

Data collection	Raw-qPCR results were obtainment with Bio-Rad CFX Manager™
Data analysis	bwa (alignment via Burrows-Wheeler transformation) v0.7.17-r1188, GATK v4.1.4.1, Eigensoft smartpca version: 16000, R version 4.0.5 (2021-03-21), ADMIXTURE Version 1.3.0, with R packages StAMPP v1.6.1, geoshpere v1.5-10, ggplot2 v3.3.3, ggpubr 0.4.0, PLINK v2.00a2.3LM. We made the maps in ggplot2 and the polygons of the Swedish counties were download from Thenmap API v2 (http://api.thenmap.net/v2/se-4/geo/2020-06-03, http://api.thenmap.net/v2/se-7/geo/2020-06-03). Further, we used Inkscape to add the arrow and improve panel spacing.

For manuscripts utilizing custom algorithms or software that are central to the research but not yet described in published literature, software must be made available to editors and reviewers. We strongly encourage code deposition in a community repository (e.g. GitHub). See the Nature Portfolio guidelines for submitting code & software for further information.

Data

Policy information about availability of data

All manuscripts must include a data availability statement. This statement should provide the following information, where applicable:

- Accession codes, unique identifiers, or web links for publicly available datasets
- A description of any restrictions on data availability
- For clinical datasets or third party data, please ensure that the statement adheres to our policy

The qPCR data and Sanger sequences that support the findings of this study are available in figshare with the identifier 10.6084/m9.figshare.14837859
The raw sequencing data that support the findings of this study are available in DDBJ DRA with the identifier DRA012297 [https://ddbj.nig.ac.jp/resource/sra-submission/DRA012297]

The Picea abies v1.0 refence genome is available here ftp://plantgenie.org/Data/ConGenIE/Picea_abies/v1.0/FASTA/GenomeAssemblies/Pabies1.0-genome.fa.gz

Field-specific reporting

Please select the one below that is the best fit for your research. If you are not sure, read the appropriate sections before making your selection.

☐ Life sciences ☐ Behavioural & social sciences ☒ Ecological, evolutionary & environmental sciences

For a reference copy of the document with all sections, see nature.com/documents/nr-reporting-summary-flat.pdf

Ecological, evolutionary & environmental sciences study design

All studies must disclose on these points even when the disclosure is negative.

Study description	We used MIG-seq sequencing to recover genome-wide SNPs in millennia-old clonal Norway spruce (Picea abies L. Karst) which might have originated from cryptic refugia in western Norway. We compared the obtained genotypes to SNP data obtained from spruce forest trees in the same areas in central Sweden. To confirm the presence of spruce in the study area during Holocene and wider areas in Fennoscandia, we used qPCR to target a mitochondrial locus which shows a haplotype unique to spruce in lake sediments and peat records covering periods of time in the Holocene and full glacial periods.
Research sample	We extracted DNA from needles of living Norway spruce (Picea abies) trees in central Sweden. These trees were sampled because they are central for answering our research questions as they are clones of ancient individuals arrived very early in the region. Further we extracted DNA from 8 threes in the central European distribution of Norway spruce, and from needles from two individuals of the sister species Picea obovata, to put the central Sweden samples into a larger perspective.<br>We also used lake sediments and peat records from Sweden, Norway, Finland, and European Russia from previously published studies. Subsamples were stored at -20C. These samples were chosen because of their availability and the sampling locations, which were relevant for investigating spruce presence in Fennoscandia and related areas.
Sampling strategy	We aimed to sample at least 20 unrelated individual trees per locality (population), as this number is thought to be a good representation of a local population, and these in our case were composed of a small number of trees scattered above the treeline. Because we sample at least 20 individuals for the clonal trees, we also aimed to sample ~20 individuals of the surrounding forest, to keep the sampling balanced. We sampled one or two small branches from healthy trees and subsequently used needles for DNA extraction. For all the trees above the tree line, coordinates were recorded with a GPS. For the ancient DNA material, we used all the lake sediments and peat records that were available. All the ancient DNA lab work was performed in an ancient DNA lab at Uppsala University. Subsampling of the cores was done in clean rooms, where no molecular lab work has been performed.
Data collection	Sampling was conducted by KN, UG , AN and LP in two times, four days in May 2019 and four days in October 2019. GPS data was collected in the field using a handhold GPS device, and transferred from the devise to a excel spread sheet by KN. The qPCR melting curves data was collected by qPCR (Bio-rad) instrument and extracted to CSV files in the CFX Manager™. The sequencing data was generated from DNA extract based on published MIG-seq protocols on an Illumina MiSeq sequencer, performed in at the Tohoku University in Japan. Sanger sequencing results were obtained from a commercial lab Macrogen (Amsterdam, Netherlands).
Timing and spatial scale	We collected data from ancient lake sediments and peat records dating back to 42 cal. kyr. BP. The spatial scale used were both regional as for the population genetic analyses, and covering the entire Fennoscandia for the ancient DNA part of the analyses. The time of coring for theses sediments ranges from 2008 to 2018 (NWF:2012; EFL:2016; NER:2012; SR:2010; SF:2010; CS-KL: 2008; CN-RD:2008; SWS:2008; SES:2015, ZF10:2018, ZF11:2018, ZF18:2018, ZF19:2018).<br>Modern spruce sampling was conducted durning 2, 4–5-day sampling expeditions in May and October 2019. DNA was extracted in 2019 and sequenced in beginning of 2020. The DNA extraction of the sediments and subsequent qPCRs were run were performed during 2018-2020 – depending on time availability of KN. No time sensitive actions were preformed, and the time of date of data collection does not have impact of the obtained results.
Data exclusions	Three samples that were sequenced were not included in the final analysis. Three extracts of the same tree were sequenced, only the sample with the most SNPs was used in the analysis. Two samples in one of the populations were very similar, potentially related, the sample with the least number of SNPs was omitted. This did not alter the results.
Reproducibility	All qPCRs performed on ancient DNA extracts were repeated 8 times to detect rare taxa, which is common in sedimentary ancient DNA. If a taxon is present in a sample, a certain number of the qPCR repeats will be positive. We used a high number of PCR controls, and extraction controls, to make sure the results were robust and to monitor for contamination during DNA extraction, PCR and library preparation. Sediment material for many of the original samples are still available and stored at -20°C and can be used in future studies to reproduce our work. The SNP analysis were performed one time, because the samples were of high quality. Frozen tissue samples are available for all sampled spruce trees, and available for future use.
Randomization	We run sedimentary samples consistently from old to young in batched of up to eight samples, restricted to a single core. We included PCR controls during all qPCR runs. We chose not to randomise samples between cores to prevent cross-contamination. For the modern DNA obtained from the spruce trees, no randomisation was preformed because genotypes are not depending on the order in which they are analysed.
Blinding	Blinding is not relevant for the study because we did not aim to test the methodology, nor are the sample names related to sensitive information. Moreover, the results are not depending on whether the sample name, location, and age are known during analysis.

Did the study involve field work? ☒ Yes ☐ No

Field work, collection and transport

Field conditions	Field conditions such as weather were not relevant for our study. The field conditions do not affect the quality or quantity of samples during collection of needles from spruce trees.
Location	Spruce needles<br>Sweden: Jämtland, Åreskutan, 63.40 N 13.05 E<br>Sweden: Dalarna, Fulufjället, 61.63 N 12.67 E<br>Sweden: Dalarna, Härjehögarna, 61.72 N 12.15 E<br>Sweden: Jämtland, Hårdeggen, 63.17 N 12.35 E<br>Sweden: Dalarna, Städjan, 61.89 N 12.71 E<br>Sweden: Dalarna, Sälen, 62.55 N 12.35 E<br>Sweden: Jämtland, Snåsahögarna, 63.22 N 12.42 E<br>Sweden: Härjedalen, Lill-Skarven, 62.55 N 12.35 E<br>Sweden: Härjedalen, Sonfjället, 62.29 N 13.57E<br>Norway: Drevfjället, 61.77 N 12.08 E
Access & import/export	The field sites were accessed by car, and sampling sites were reached on foot. Samples were stored in plastic bags with silica gel and transported in cold conditions to the University laboratories by car. They were not required to be imported or exported from different countries.
Disturbance	No disturbance occurred.

Reporting for specific materials, systems and methods

We require information from authors about some types of materials, experimental systems and methods used in many studies. Here, indicate whether each material, system or method listed is relevant to your study. If you are not sure if a list item applies to your research, read the appropriate section before selecting a response.

Materials & experimental systems

n/a	Involved in the study	
☒	☐	Antibodies
☒	☐	Eukaryotic cell lines
☐	☒	Palaeontology and archaeology
☒	☐	Animals and other organisms
☒	☐	Human research participants
☒	☐	Clinical data
☒	☐	Dual use research of concern

Methods

n/a	Involved in the study	
☒	☐	ChIP-seq
☒	☐	Flow cytometry
☒	☐	MRI-based neuroimaging

Palaeontology and Archaeology

Specimen provenance	SWS, Sweden, 56.28N, 12.85E, Lake/peat<br>SES, Sweden, 56.27N, 15.02E, Lake/peat<br>SF, Finland, 61.15N, 24.67E, Peat<br>SR, Russia, 57.10N, 32.75E, Lake, Lake<br>NWF, Finland, 68.15N, 25.62E, Fluvial peat<br>NER, Russia, 67.03N, 62.55E, Lake<br>Salla, Finland, 66.5N, 28.40E, Fluvial peat<br>Kou, Finland, 24.8N, 66E, Fluvial peat<br>KL, Sweden, 63.29N, 12.48E, Lake<br>ZF10, Sweden, 63.62N, 12.26E, Lake<br>ZF11, Sweden, 63.62N, 12.25E, Lake<br>ZF18, Sweden, 63.31N, 12.90E, Lake<br>ZF19, Sweden, 63.31N, 12.91E, Lake<br>RD, Norway, 63.37N, 11.82E, Lake
Specimen deposition	Material for which material remained after extraction are stored at Uppsala University
Dating methods	Dating for most sites have been published in peer reviewed journals as specified in the manuscript. For few samples, new AMS radiocarbon 14 dating was used, see supporting information.

☒ Tick this box to confirm that the raw and calibrated dates are available in the paper or in Supplementary Information.

Ethics oversight	No ethical approval is required for working with lake sediments and peat records

Note that full information on the approval of the study protocol must also be provided in the manuscript.
